# Themes of advanced information processing in the primate brain

**DOI:** 10.3934/Neuroscience.2020023

**Published:** 2020-10-15

**Authors:** Robert Friedman

**Affiliations:** Department of Biological Sciences, University of South Carolina, Columbia 29208, USA

**Keywords:** information encoding, primate brain, cerebral cortex, perception, visual processing

## Abstract

Here is a review of several empirical examples of information processing that occur in the primate cerebral cortex. These include visual processing, object identification and perception, information encoding, and memory. Also, there is a discussion of the higher scale neural organization, mainly theoretical, which suggests hypotheses on how the brain internally represents objects. Altogether they support the general attributes of the mechanisms of brain computation, such as efficiency, resiliency, data compression, and a modularization of neural function and their pathways. Moreover, the specific neural encoding schemes are expectedly stochastic, abstract and not easily decoded by theoretical or empirical approaches.

## 1. Introduction

The mammalian brain is unique in its organization, function and structure. The neurons and their connections are organized in a way that allow for complex processing and storage of information from neural input sources [1]–[6]. The inputs may originate from a sensory system or from within the brain itself. The internal cortical processes of the brain operate on a flow of abstract information, otherwise known as mental representations, with a relatively weak connection to actual objects of the external world [7]–[17]. Currently, there are established examples of brain computation that provide a template on how neural systems are expected to function and the biological constraints on these systems. Moreover, they provide insight for artificial design, such as in simulation of these neural pathways [18]–[19]. Likewise, models devised by engineers serve to help validate the efficiency of neural systems in nature [20]–[23].

Neuroscientific studies have approached these questions at different scales and perspectives. One is from the field of perception and our ability to perceive the external world [24]–[30] while another strives to unravel the molecular mechanisms of neural function in the brain [14],[16],[31]–[34]. The neurobiological perspective includes the structure and function of the brain regions and scales upward to the system level processes. For example, as much as one-half of the cerebral cortex is dedicated to visual processing and is anatomically structured as a series of layers. The primary visual cortex further divides into two anatomical pathways known as the dorsal and ventral streams [35]–[36]. The organization of these and other downstream regions is hierarchical in nature. The neurobiology of the other senses are relatively less studied, but they show analogous processes to those found in vision [37].

In reference to the field of perception, studies have established the theory of constancy which predicts that sensory stimuli are neurally processed for buffering against the effects of changes in orientation, time and other attributes that effect how a stimulus is received [13],[25],[38]–[44]. This theory requires a basis for object identification, whether visual in type or from another sensory modality, and an encoding scheme within the neural structure of the cerebral cortex [9],[13],[45]–[49]. These cortical regions further interact with regions involved in memory storage which is a participant in the formation of mental representations [31],[33],[50]–[51].

Even though our perception of the external world is typically in three dimensions, the actual sensory interface acquires data in two dimensions [9]–[10],[22],[52]. For example, the visual information is initially projected in 2d onto an eye's retina. A three dimensional perspective of the external world, so that depth is realized, is later reconstructed in the cerebral cortex by a substantial amount of image processing across different cortical layers [53]–[54].

**Figure 1.**
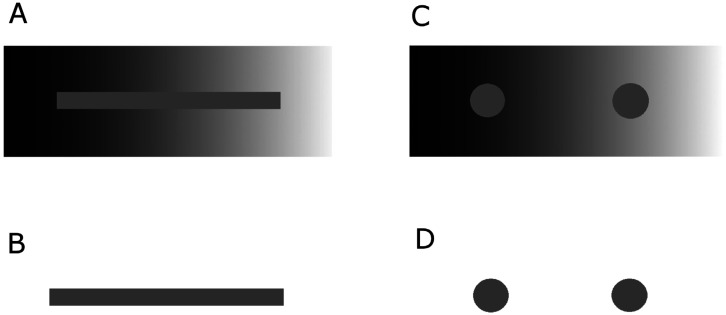
Examples of brightness constancy in vision. A: A horizontal rectangular shaped bar is pasted within the larger background of a gray color gradient scheme. The background color scheme is perceived correctly, but the bar is actually a single color of gray even though it appears to have a gradient of gray colors. B: The identical bar from above is shown without a background of color to prove that the bar is actually a single gray color. C: This figure shows a similar visual effect as in 1A except that two circle shapes are plotted against the background instead of a rectangular bar. As in 1A, the color shapes are perceived as different colors of gray even though they are the same gray color. D: The identical circle shapes from above are shown without a background of color to prove that the circle shapes are in fact the same gray color.

Visual processing includes adjustment to the attributes of emitted light, including that of brightness and contrast [25],[40],[42], color [41],[44], size [39],[55], and motion [56]. The theory of visual constancy also requires that these attributes are invariant, and therefore resistant, to modification from changes in the background and the environment [43]. An example of brightness constancy is shown in Figure 1. Without this generation of invariance in the cortical modeling of objects, the data is insufficient to identify objects and acquire knowledge of the external world [39],[57].

The above examples, and the structure and function of the cerebral cortex, originate from the processes of animal development and evolution [58]. These constrain the form of the neural network and its efficiency in solving the problems of information flow across the brain and the sensory systems. For example, there is evidence that a sensory system is overly connected to its target in the cerebrum during development in humans, such as the case where there is a peak in perception for replicating the elements of speech, but disuse of particular phonemes leads to their irreparable loss [59]–[61].

This review focuses on empirical examples of advanced information processing in primates. Included are those from visual processing, object identification and perception, information encoding, and memory. These processes are considered essential for developing artificial models of cognition in primates. Toward this goal, well established examples that are representative of a cognitive scheme are included. However, this review generally excluded: the large number of evolutionary comparisons from the cognitive sciences, a comprehensive search of all examples per process, and models without empirical support. The intention here is that the current knowledge of cognition is synthesized as general themes that are established and informative for developing a perspective of large scale processes of the brain.

## 2. Visual processes in general

Vision requires extensive processing by the brain of primates and other non-primate vertebrates [13],[43]. Evidence of this ranges from studies of perception to those of neurobiology [25],[34],[62]–[63]. Work in perception has led to the theory of visual constancy which predicts that incoming visual stimuli is selected for maximizing for the identification of objects [13],[43]. This is also required by downstream processes of visual data, otherwise the receiver is expectedly left with a deficit in perception of the external world [57].

The visual data originates as a two dimensional image across the retina of the eye [52]. This information is further processed along the many layers of the cerebral cortex, and travels along two pathways of the primary visual cortex, the ventral and dorsal streams [35],[64]. Studies have revealed some of the workings of this system, including the cortical subregions involved in facial recognition which is likely a generalized process for identification of visual objects [47],[62],[65]. It is also likely that these schemes are the same in auditory and other sensory systems that target regions of the cerebral cortex.

Common themes in brain function are supported by studies of neonatal ferrets which develop vision even though the optic nerve is rewired to the auditory center of the cerebral cortex [66]–[67]. They investigated the animal's capacity for selecting direction and orientation in their visual environment. After training of the rewired ferrets, the auditory cortex developed a response to visual stimuli and formed an approximate map of neural connections to that of a normal visual cortex. These studies show a bottom-up process of neural development and structure that is dependent on sensory input [48]. Moreover, it is possible that the auditory and visual cortices are homologous, possibly by duplication over evolutionary time, and share the same potential for three dimensional mapping and reconstruction of the external world [68]–[69].

A similar result was observed in humans. An individual born with only a left hemisphere of the cerebral cortex developed a map of the visual field for the left eye, but also compensated by developing a map in that hemisphere for the right eye [70]. These studies illustrate a high resiliency to error during the development of the cerebral cortex.

There is also evidence of convergent evolution at the level of the neural circuitry and suggests an unavoidable optimal design by nature. An example is from the mechanism of motion detection across the visual field of *Drosophila* [71]–[72] and hoverflies [73]. These insects have a compound eye that independently evolved from the camera eye of mammals, yet the arrangement of neurons and their connections are similar for processing motion detection.

## 3. Object identification and facial recognition

Object recognition in general, such as for non-face objects, is likely a similar process as for the better studied recognition of faces [8],[14],[16],[45]–[47],[49]. Face recognition in primates is recognized as a sequence of separate and hierarchical visual processes, is fairly resilient to error, and the process generally occurs within a few hundred milliseconds [9],[46],[48],[74]–[78]. This process has been studied by a focus on the cerebral cortical regions that range between the early visual cortex and the ventral stream [45]. The ventral stream targets the temporal region of the cerebral cortex while the dorsal stream targets the parietal lobe. These two paths are not considered perfectly distinct according to their neural function, but in general the dorsal path is associated with a spatial and temporal mapping of visual objects. The ventral path is involved in object recognition by feature detection [14],[45],[48],[78].

Constancy and invariance of the initial visual stimuli is a requirement for object identification that occurs in the later stages of image processing [8],[25],[39],[43],[65]. This statement is supported by a quote from Freiwald and Tsao [8]: “The greatest obstacle to object recognition is the huge amount of variation that can occur in the retinal images cast by a 3D object.” This problem is a result of the physical limitations involved in projecting a 3d image onto the 2d retinal surface of the eye. Therefore, it is inferred that naive visual processing is not invariant to error, so the evolution of visual constancy is required for reconstructing objects in the visual field that reflects the external world. This advanced form of visual processing is dependent on natural selection for robustness to visual perspective and a compression of information to fewer dimensions. Adaptations for reconstructing a 3d image of the world also includes binocular vision and perception of surface textures [52],[54]. For example, the texture cue is active in all cases, even in the monocular case.

For both the ventral and dorsal streams, the image travels first from the retina to a region of the thalamus where processing includes a correction for variability in brightness and contrast [9],[79]. The next step in the pathway is the early visual cortex before separating into ventral and dorsal streams. This area has a neuron count that is about two orders of magnitude greater than the earlier steps of the pathway. The early visual cortex encodes the raw visual data and includes advanced but imperfect processing that is revealed by the line-motion illusion experiment. This processing also shows enhancements by the capability to discriminate a set of visual patterns that vary in contrast against a background [80]–[81]. Altogether, these examples show that a reconstructed image is correlated with the perception of an image rather than the physical image itself.

Chang and Tsao [14] further investigated populations of neurons in the ventral stream of the visual cortex. In particular, they examined an early step in this pathway with about 200 neurons that are involved in the identification of facial objects. They showed that this neuron set codes for a set of linearly combined metrics of facial features which supports a linear axis based model of feature sampling. They rejected the alternative hypothesis that each face is encoded by a set of non-linear distance based metrics which represent differences between the observed and expected face of interest. They considered their finding as a probable explanation for the encoding of other kinds of objects, particularly since their neural model shows a general resilience to error where the viewer observed an object at different orientations.

## 4. Memory encoding and spatial navigation in the hippocampus and entorhinal cortex

The hippocampus includes the function to map spatial relationships, specifically there is evidence these maps in mammals are used for memory encoding and in spatial navigation [7],[50]–[51],[82]–[86]. The spatial mapping function involves place cells and likely grid, head direction, and border cells of the hippocampal-entorhinal circuit [33]. Buzsaki and Moser [31] suggested that memory and navigation are mechanistically homologous, that the paths and landmark-based maps involved in navigation are analogous with memory that is coded as a path and map of a sequence of events and objects. This hypothesis is supported by Constantinescu and others [11] and is a reasonable explanation given that the internal algorithm for spatial mapping is generalizable in function beyond the obvious use in navigation, such as for associating words with perceived objects of the sensory systems.

**Figure 2.**
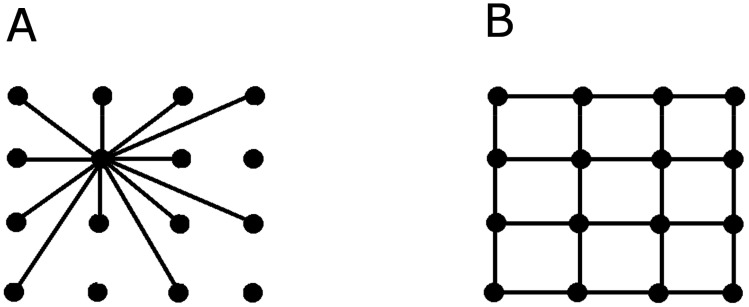
Two different topologies of network organization. A: A small-world network [87] that is considered the null hypothesis for how the neural system is organized in the brain. B: An alternative hypothesis of a lattice like network that fits with the two dimensional pattern of regularly arranged neurons that code for spatial maps.

These spatial maps have been localized to a 2d array of specialized neurons and their interconnections, such as the place and grid cells. In particular, the place cells are thought to code for an animal's spatial location in conjunction with current or past events [33]. However, the precise encoding scheme is unknown. Likewise, the grid cells represent a map of the external environment and there is a suggestion that they are organized into modules with the possibility for the generation of thousands of these spatial maps [33]. The arrangement of these cells fits with a lattice like structure in their spatial organization and regularity. An example of a lattice like structure is shown in Figure 2. The question is whether it is possible to detect these arrangements by current algorithms.

## 5. Visual attention in primates

Visual attention is a process that is considered a synergism of brain computation, such as memory, recognition of objects, and a map of spatial relationships [13],[88]–[95]. The literature defines the major prerequisites of the visual attention model: perceptual salience—robust identification of important features of a visual scene; object recognition; explicit map for neural coding of saliency; resilience to disturbance in visual attention; and adaptation of eye and body motion for fovea based vision of salient features. Likewise, the dynamics of visual attention are considered dependent on factors such as the visual search task, bottom-up and top-down cues, and maintenance of foveal visual acuity. An example of a bottom-up cue is the visual contrast of a feature while a top-down cue may be a weight assigned to the importance of a feature. Altogether these processes combine simple and complex processing of visual information. Some of these processes occur over a short time scale, <100 milliseconds, while others occur over a longer time scale which are adapted for a top-down process involving recurrent neural systems [91].

It is possible to consider visual attention as a mechanism for maximizing the extraction of important data from around 10^8^ bits of visual information that flow across the optic nerve per second [90]. Visual attention expectedly leads to awareness of an object in a given visual scene, but this recollection is reducible to the neural mechanisms of cognition. This process is also expected to contribute to the constancy of visual stimuli and therefore reduction of a high dimensional sensory input to a simpler form for the downstream cognitive processes.

## 6. Hypotheses to describe information encoding across the cerebral cortex

**Figure 3.**
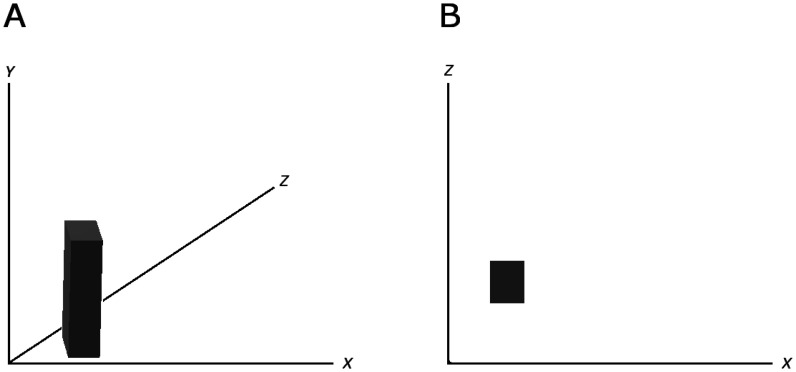
Example of dimension reduction of a 3d rectangular box to a 2d rectangular shape. A: The rectangular box plotted in three dimensions. B: The 3d box from 2A is reduced to two dimensions so only the 2d rectangular shape is observed. In this case, the information on height of the box is lost.

There are theories on how information is encoded across the brain [13]. One model has the expectation that data is handled by populations of neurons, not by individual cells. Another prediction is that the neural code is compressible, such as by dimension reduction where a set of correlated variables is reduced to an uncorrelated set, given that the high dimensional external world is reliably captured by fewer dimensions [96]. An example of dimension reduction is shown in Figure 3. Other hypotheses on brain computation are available from modeling information flow in the framework of a dynamical system of neurons [15],[17],[22],[34],[97]–[98].

Empirical work has supported the above theories. A study of the primate prefrontal cortex revealed an encoding of memories for sequences of objects that is both heterogeneous and disordered [32]. They also established that this finding of “mixed-selectivity of neuron responses” is consistent with coding that is high dimensional and that any reduction in dimensionality of the code leads to error in the encoding process. This is hypothesized as a mechanism for the requirements of a generalized response to information, but also that highly specialized neurons are expected to instead encode at a level of low dimensionality [12]. These findings are related to the problem of efficiency in biological computation and a reason to pursue theory to find the constraints on an information based system, such as that offered by the dynamical system and network approaches [13],[99]–[103]. One theoretical approach was undertaken by Yang and others [23] where they trained an artificial neural network (ANN) to a set of cognitive tasks. They found that the resulting network structure included artificial neurons that had “mixed task selectivity” as was previously observed in experiments by Rigotti and others [32]. Others have also pursued this approach to find brain-like structures among ANNs [104]–[106]. ANNs are thoroughly reviewed by Kriegeskorte [104].

The more traditional hypothesis of neural coding efficiency predicts that the encoding process by neurons is selected for information flow efficiency where the data is uncorrelated and higher dimensional; while selecting for robustness of information transmission where the data is correlated and lower dimensional [107]. The latter idea fits with object identification where a major factor in data transmission is in the accuracy of reconstructing an object in the visual field, and that the appearance of the object is robust to problems such as a noisy visual background [108]–[110]. Higher informational processes, such as for mental representation, instead fit with the predictions for coding at maximal efficiency [111]–[112].

## 7. Predictive coding of sensory information

Both vision and speech are processed in real-time, but there is a delay based on the time for brain computation [113]–[120]. This typically occurs at the millisecond scale [113]. The problem is that the time delay is too long to adequately respond to the external world, such as to evoke a motor response for avoiding an object [118]. The brain compensates for this delay by predictive coding, such as in constructing the location of a visual object from prior knowledge of its speed and direction. This compensatory process presumably applies to all other sensory modalities.

The temporal aspect of perception further reinforces the concept that the internal representation of the external world is a reflection of the raw input that is initially received by the senses. Further, the time delay for processing of real-time sensory data, not only for the goal of object detection, but also for the cognitive processes that are a hallmark of the human experience [116]. A hypothetical example is where visual images are cognitively processed alongside stored memories, the time for this advanced processing compensated by predictive coding. A reported example is in the case of semantic modeling of speech so that the listener's response is rapid [119]–[120]. There are similar studies of predictive coding in the case of musical syntax [114],[117].

## 8. Time delay in perception of a motor action

A related phenomena of time delay in perception is observed in studies that measure the delay between perceptual experience of a motor action and the voluntary motor movement itself [116],[121]–[127]. The perceptual experience is reported after the neurons involved in the motor action have fired. This time delay has been interpreted as a perceptual experience that favors compression of time between the voluntary motor action and the outcome of that action [127]. Overall, this phenomenon could be interpreted as another example of reduced complexity of input from the external world. This may be restated as a favoring for invariance of temporally variable experiences.

The above investigations are also concerned about perceptual awareness of these motor actions as occurring many milliseconds after the action itself. There is an additional effort to establish terminology that has a testable and mechanistic basis that is not intertwined with transcendental thinking [127]. These two elements have led to the theory that an action is the cause of a perceptual awareness instead of the traditional expectation that our thought is the cause of an action [128]. In either case, there is evidence here that the temporal perception of a motor action is adapted to the processes of cognition.

## 9. Conclusion

Themes for understanding information encoding in the brain include modularization of neural structure and function, encoding at the population level of neurons, resiliency to error across paths in the neural pathway, compensation for processing time, and efficiency of data transmission along with compression. In addition, there are populations of neurons that serve a general function while others are specialized, and that this leads to predictions on the neural dynamics across space and time. The data encoding schemes are likely in abstract form [129], especially further along an information processing pathway, and suggest that the mechanisms of mental representation occur in a neural language that is not easily relatable to the perceived mental state. The advanced visual processes support these concepts, such as the adaptation of visual objects for information transmission efficiency and perception of the external world, but not as a means to reconstruct a fully accurate and objective representation of objects.

The visual system is better understood along with examples of specialized neural populations and their functions. One reason is that the properties of light are simpler to model by the mathematics of optics theory while the studies of how objects are internally represented is not easily superimposed onto neural systems and their pathways. However, it seems certain that in general these complex and evolved neural systems are efficient in their information handling and resilient and robust to error [130]. Also, the noisy nature of sensory stimuli is highly compressible and that the corresponding neural pathways are resilient to error in reconstructing the external world, while the mental representations are relatively uncompressed and that the coding process is heterogeneous and disordered [12]. These findings altogether provide expectations on the dynamics of information flow across the cerebral cortex, a higher scale process that mirrors a communication system that is undergoing a continual birth and death process of links in the network [98],[131]–[132].
